# Liquid Phase Plasma Synthesis of Iron Oxide Nanoparticles on Nitrogen-Doped Activated Carbon Resulting in Nanocomposite for Supercapacitor Applications

**DOI:** 10.3390/nano8040190

**Published:** 2018-03-25

**Authors:** Heon Lee, Won-June Lee, Young-Kwon Park, Seo Jin Ki, Byung-Joo Kim, Sang-Chul Jung

**Affiliations:** 1 Department of Environmental Engineering, Sunchon National University, Suncheon 57922, Korea; honylee@hanmail.net (H.L.); lone0486@naver.com (W.-J.L.); 2 School of Environmental Engineering, University of Seoul, Seoul 02504, Korea; catalica@uos.ac.kr; 3 Department of Environmental Engineering, Gyeongnam National University of Science and Technology, Jinju 52725, Korea; seojinki@gmail.com; 4 R&D Division, Korea Institute of Carbon Convergence Technology, Jeonju 54853, Korea; kimbj2015@gmail.com

**Keywords:** liquid phase plasma, activated carbon powder, iron oxide nanoparticle, nitrogen-doped carbon, pseudo-capacitive characteristics

## Abstract

Iron oxide nanoparticles supported on nitrogen-doped activated carbon powder were synthesized using an innovative plasma-in-liquid method, called the liquid phase plasma (LPP) method. Nitrogen-doped carbon (NC) was prepared by a primary LPP reaction using an ammonium chloride reactant solution, and an iron oxide/NC composite (IONCC) was prepared by a secondary LPP reaction using an iron chloride reactant solution. The nitrogen component at 3.77 at. % formed uniformly over the activated carbon (AC) surface after a 1 h LPP reaction. Iron oxide nanoparticles, 40~100 nm in size, were impregnated homogeneously over the NC surface after the LPP reaction, and were identified as Fe_3_O_4_ by X-ray photoelectron spectroscopy and X-ray diffraction. NC and IONCCs exhibited pseudo-capacitive characteristics, and their specific capacitance and cycling stability were superior to those of bare AC. The nitrogen content on the NC surface increased the compatibility and charge transfer rate, and the composites containing iron oxide exhibited a lower equivalent series resistance.

## 1. Introduction

Recently, the hybrid electric vehicles (HEV) and plug-in electric vehicles (PEV) have attracted attention and are showing rapid growth [1,2,3]. HEV and PEV require a low-resistance, high-voltage secondary battery and a supercapacitor [4,5]. Supercapacitors have received a great deal of interest because of their higher power densities compared to batteries and higher energy densities than conventional capacitors [6,7].

The capacitance of a supercapacitor can be changed according to the electrode material [8,9]. Therefore, there has been considerable interest in discovering ideal materials for supercapacitor electrodes with respect to different metal oxides [10,11] and carbonaceous materials [12,13]. Among the metal oxides investigated thus far, iron oxide is considered one of the most attractive because it displays high theoretical capacity, and is inexpensive [14,15,16]. Carbonaceous materials, particularly activated carbon (AC), are the most widely used electrode materials of electrochemical double layer capacitors (EDLCs) because of their large specific surface area, size adjustment of pores, chemical stability, lower weight, low cost, excellent electrical conductivity, and environmental friendliness [17,18]. Many researchers have prepared carbon electrode materials with a high specific capacitance for EDLCs [19,20]. The introduction of electronic conductivity by doping the carbon matrix with nitrogen is a promising alternative to enhancing the specific surface area [21,22]. Two methods for nitrogen doping have been reported: carbonization of abundant nitrogen such as polypyrrole from nitrogen-containing precursors [23,24] and introducing nitrogen-containing reagents such as ammonia, urea, amines, and melamine to the carbon matrix [25,26,27]. Recently, the plasma generated from the liquid phase has been used to synthesize metal oxide nanoparticles, which were then impregnated with carbonaceous materials [28,29,30]. In previous studies, carbon composites impregnated with metal oxide nanoparticles were synthesized using a liquid phase plasma (LPP) method and applied to EDLCs [31,32,33]. In particular, the LPP method does not require the use of any additional reducing agent, and is a very simple process for producing composites in a single step [29,34].

In this study, the LPP method was used to dope nitrogen and impregnate iron oxide nanoparticles on the AC surface. The composition, shape, and chemical state of the fabricated composite produced from the LPP reaction was quantitatively and qualitatively analyzed by various instruments. Also, the effects of the amounts of nitrogen loaded and iron oxide particles impregnated on the AC surface on the electrochemical performance for the prepared composites were investigated in detail. Specifically, the main research questions of this research are (1) whether the specific capacitance of the prepared composite increased with increasing quantity of iron oxide precipitate or not, and (2) whether the composite showed resistance with the highest initial resistance slope or not.

## 2. Materials and Methods

### 2.1. Materials and Chemicals

Activated carbon powder (YP-50F, Kuraray Chemical Co. Ltd., Osaka, Japan) was used as the electrode active material. Ammonium chloride (NH_4_Cl, Daejung Chemicals & Metals Co., Siheung, Republic of Korea) was used for doping the activated carbon powder with nitrogen. We specifically selected ammonium chloride that were an alkaline substance with high solubility in water, as compared to urea, glycine, nitric acid, etc., to minimize the effect of precursor type and concentration on the LPP process in terms of both conductivity and pH. However, a significant reduction of pH was still observed in the reactant solution after the LPP reaction. Nitrogen-doped activated carbon powder was subjected to the LPP reaction to impregnate iron oxide nanoparticles using iron chloride tetrahydrate (FeCl_2_·4H_2_O, Kanto Chemical, Tokyo, Japan) as the precursor. Cetrimonium bromide (CTAB, CH_3_(CH_2_)_15_N(Br)(CH_3_)_3_, Sigma-Aldrich, St. Louis, MO, USA) was added to the LPP reactant solution to disperse iron oxide nanoparticles on the surface of nitrogen doped activated carbon. All chemicals used in this study were reagent-grade chemicals and ultra-pure water (Biological industries, Beit, Israel) was used to prepare the aqueous LPP reaction solutions.

### 2.2. Experimental Device

The LPP system was used in the same process for the nitrogen doping on the surface of AC or impregnating iron oxide nanoparticles on nitrogen doped activated carbon. The LPP device consisted of a power generator, a reaction unit, and a cooling system. Details of the configuration and specifications of the LPP device are reported elsewhere [31,32]. In both processes, the plasma operating conditions were a frequency of 30 kHz, a pulse width of 5 µs, and an applied voltage of 250 V. The quartz LPP reactor is a double tube type with an outer diameter and height of 40 and 80 mm, respectively. The batch type reactor was filled with the reaction aqueous solution, and the outer channel was circulated with cooling water at 268 K. The cooling system played an important role in eliminating the effect of temperature, which is highly elevated during the LPP reaction due to energy released by arc plasma in the reactant solution on the generated nanoparticles. The spacing of the tungsten electrodes embedded in the ceramic insulators was maintained at 1.0 mm.

### 2.3. Preparation of Nitrogen Doped Carbon

Nitrogen doped carbon (NC) was synthesized through the LPP reaction. NH_4_Cl, as the nitrogen precursor, was added to 200 mL of ultrapure water to make a 10 mM solution and 0.5 g of AC was then added and stirred for 30 min to prepare the LPP reaction aqueous solution. Plasma was generated in this aqueous reaction solution for 60 min to dope the AC with nitrogen. This is because both the amount of iron oxide nanoparticles generated and the efficiency of nitrogen doped carbon are increased only up to 60 min of the LPP reaction. After the LPP reaction, the NC powder was centrifuged and washed three times with ultrapure water to remove the impurities. Finally, NC was prepared by drying in a vacuum oven at 353 K for 24 h.

### 2.4. Preparation of IONCC

Iron oxide nanoparticles were impregnated into NC using the LPP reaction. A 0.5 g sample of NC powder prepared by the LPP reaction was added to 200 mL of ultrapure water containing CTAB and dispersed by stirring. Iron chloride (5 and 10 mM) was then added to the reactant solution, which was then dissolved by stirring for 10 min. Plasma was generated for 60 min to prepare the NC composite impregnated with iron oxide nanoparticles. The resulting iron oxide/NC composite (IONCC) powder was washed and dried using the same method for NC production.

### 2.5. Electrochemical Test

A half-coin cell was prepared using IONCC powders and its electrical properties as a supercapacitor electrode were assessed. The slurry for the coin cell was prepared by dissolving the active material (80%), conductive agent (acetylene black, 10%), and binder (polyvinylidene fluoride, 10%) in a *N*-methyl pyrrolidinone solvent. The active material:conductive agent:binder were mixed at a ratio of 80:10:10 wt. % to prepare the slurry. Here, IONCC powders, super-P (TIMCAL graphite & carbon com., Bironico, Switzerland), and polyvinylidene fluoride were used as the active material, conductive agent, and binder, respectively. The slurry was coated on Ni foil and dried for 60 min in a convection oven at 393 K. The resulting powder was then roll-pressed and dried in a vacuum oven at 373 K for 60 min to produce a half-coin cell. The electrolyte was a 6M KOH solution and the separator was 150 µm glass felt. The current-voltage (C–V) curve was measured at a scan rate of 10 mV/s, and actuation voltage from 0.1 to 0.8 V. All electrochemical properties were measured using VSP potentiostat (Bio-logic Science Instruments, Seyssinet-Pariset, France). Three coin cells were prepared under the same conditions, and the electrochemical properties reported are the mean values.

### 2.6. Structural Characterization

The elemental composition and dispersity of the as-prepared NC composite and IONCC were examined by field-emission scanning electron microscopy (FE-SEM, JSM-7100F, JEOL, Tokyo, Japan). The size and shape of the iron oxide nanoparticles in the IONCC were observed by high resolution field emission transmission electron microscopy (HR-FETEM, JEM-2100F, JEOL, Tokyo, Japan). The chemical structures of NC and IONCC were analyzed by X-photoelectron spectroscopy (XPS, Multilab 2000 system, Thermo Fisher Scientific, Waltham, MA, USA) and X-ray diffraction (XRD, XRD-7000, SHIMADZU Corp., Kyoto, Japan). The specific surface area and pore size distribution of the IONCC were examined using a surface area analyzer (Belsorp mini II, MicrotracBEL Corp., Osaka, Japan).

## 3. Results and Discussion

### 3.1. Characteristics of IONCC

**Figure 1:**
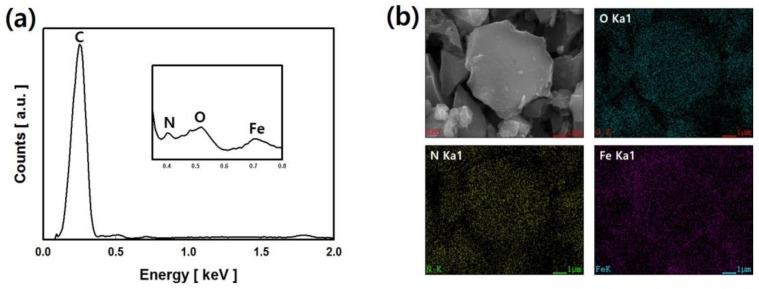
EDS spectrum (**a**); SEM image and element-mapping images; (**b**) of IONCC prepared by two LPP reactions.

The chemical composition and dispersibility of the IONCCs prepared by the LPP process were measured by energy dispersive spectroscopy (EDS) and element-mapping attached to the FE-SEM. Figure 1 presents the EDS spectrum, real image, and the mapping image of each chemical component of the IONCC produced by the LPP process.

The sample was prepared by first fabricating a NC by the LPP reaction in an aqueous ammonium chloride solution, followed by a second LPP reaction at an iron chloride concentration of 10 mM. A strong peak of carbon was observed at 0.25 keV in the EDS spectrum. Doped nitrogen (N kα) was observed at 0.39 keV after the first LPP reaction, and iron (Fe Kα) impregnated was noted at 0.77 keV after the second LPP reaction. In the mapping images, oxygen, nitrogen, and iron were marked with green dots, yellow dots, and purple dots, respectively. The mapping image revealed nitrogen and iron well dispersed over the AC surface. These results suggest that AC is doped with nitrogen by the plasma generated in the ammonium chloride solution. The iron nanoparticles adhered to the AC surface by the LPP reaction.

Table 1 lists the chemical compositions of NC and IONCCs prepared by the LPP reactions determined by EDS. The chemical composition of bare AC (YP-50F) used as an electrode active material in this study was composed of 97.06% carbon and 2.29% oxygen (at. %). Approximately 3% oxygen was detected from the oxygen-containing functional groups attached to the AC surface. Oxygen-containing functional groups are formed in the acid treatment during the production of AC. On the other hand, the nitrogen component at 3.77 at. % was detected in NC. Therefore, a nitrogen-containing functional group is formed on the AC surface when the LPP process is performed for 60 min in an aqueous ammonium chloride solution containing AC. The IONCCs were prepared by varying the initial iron chloride concentration to 5 mM (IONCC-5) and 10 mM (IONCC-10), respectively. The iron content of IONCC-5 and IONCC-10 was 0.51 and 0.89 at. %, respectively. The quantity of iron nanoparticles impregnated in the AC surface increased with increasing initial iron chloride concentration. Generating plasma in the liquid phase is usually based on a streamer discharge or spark discharge. When spark discharge occurs in the liquid phase, a large quantity of electrons, ozone and oxygen bubbles, strong ultraviolet rays, various free radicals, and over-pressure shock waves are generated [35]. Therefore, ammonium ions (NH_4_^+^) are reduced by the many electrons generated in the reaction solution to form nitrogen-containing functional groups on the AC surface. In addition, the iron ions are also reduced by these electrons and imprinted on the AC surface. The amount of oxygen contained in the NC was higher than that in bare AC, which means that AC was oxidized by the LPP reaction. In addition, the amount of oxygen contained in the IONCC was higher than that of NC. Therefore, NC was oxidized by the LPP reaction, in which iron nanoparticles were impregnated. When plasma is generated in the liquid phase, it can instantaneously generate a strong electric field, which produces a range of active chemical species (O_2_^−^, ^1^O_2_, O*, O_3_, OH*, HO_2_, H_2_O_2_, etc.) [36,37]. The AC and NC were assumed to be oxidized by these strong oxidizing active species generated in the LPP reaction aqueous solution to increase the oxygen content. On the other hand, the oxygen content of IONCC-10 synthesized at high initial iron precursor concentrations was higher than that of IONCC-5. This was caused by the impregnation reaction of iron nanoparticles, and it was presumed that iron oxide nanoparticles are formed in the LPP reaction of this study.

**Figure 2:**
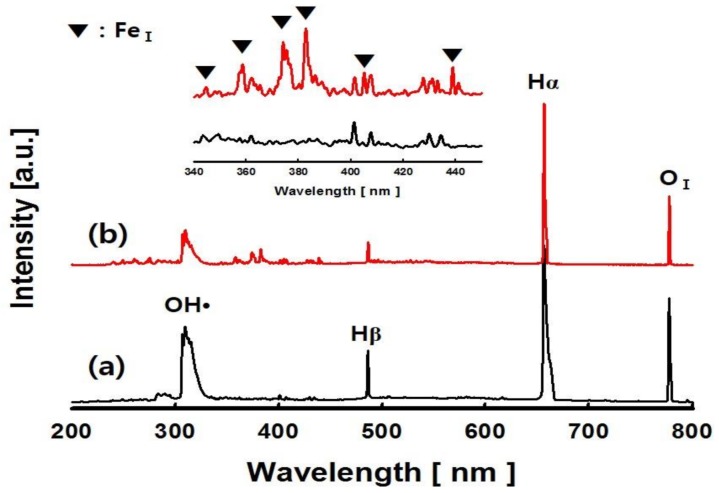
Spatially and temporally integrated emission spectra for the pulsed electric discharge. (**a**) ultrapure water, (**b**) IONCC-10 reactant solution.

Various chemically active species can be formed in the plasma field provided to the reactant aqueous solution. In this study, the chemically active species were characterized by optical emission spectroscopy (OES, AvaSpec-3648, Avantes, Apeldoorn, the Netherlands). Figure 2 shows the spectra emitted from the ultrapure water and IONCC-10 LPP reactant aqueous solution. In ultrapure water, molecular bands of hydroxyl radicals (OH^•^) with the excited states of atomic H and atomic O were observed in the emission spectrum [38,39]. In the reactant solution of IONCC-10, iron peaks were newly observed in the 340 to 440 nm regions along with those chemical species observed in ultrapure water. They revealed atomic iron (Fe_I_ ground state electron configuration 1s^2^2s^2^2p^6^3s^2^3p^6^3d^6^4s^2^, ^5^D^4^) peaks at 344.0, 358.1, 373.7, 382.0, 404.5, and 438.3 nm [40]. The LPP process can produce powerful plasma instantaneously and releases strong electric fields that generate numerous electrochemical species [35]. In addition, a very rapid reaction is followed by the active species and radicals generated under the high temperature of the LPP process cause very rapid reactions [36]. The LPP reaction applied in this study is caused by electrons and these active species. Iron oxide particles are formed by reacting charged species (i.e., ions) from iron precursor in the reactant solution with active species generated from the LPP process.

**Figure 3:**
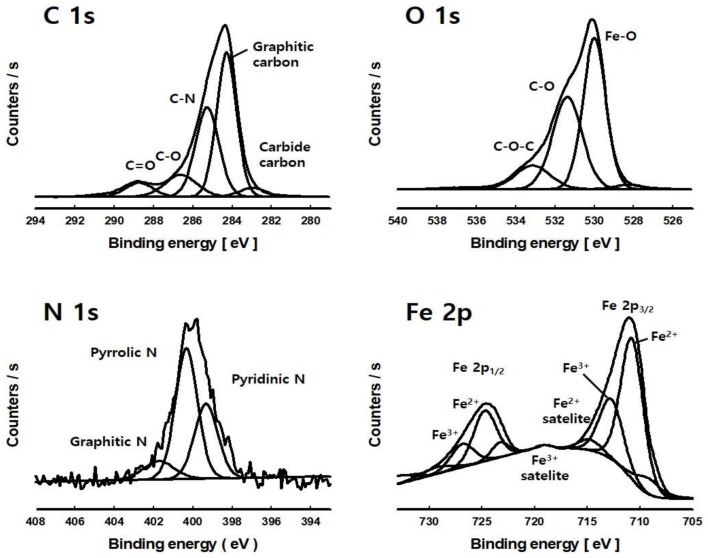
High resolution XPS spectra of C 1s, O 1s, N 1s, and Fe 2p region of IONCC-10 prepared by the LPP method.

XPS was performed to examine the chemical state and structure of nitrogen and iron particles of the IONCC synthesized by the LPP method. Figure 3 shows the high resolution narrow-range XPS spectrum of carbon, oxygen, nitrogen, and iron of IONCC-10. In the C 1s region, peaks were observed at 282.9, 284.3, 285.2, 286.6, and 288.7 eV, which were assigned to carbide carbon, graphitic carbon, C=N, C–O, and C=O bonds, respectively [41,42]. These results show that ammonium in the reactant aqueous solution reacts with AC to form a C=N bond by the LPP reaction. The observed C–O and C=O bonds can be attributed to an approximately 3% carbon content originally contained in the AC, or oxidized AC surface by the excited oxygen and hydroxyl radicals produced by the LPP process. In the O 1s region, a large Fe–O peak was observed at 530.0 eV and peaks due to C–O and C–O–C were observed at 531.4 and 533.2 eV, respectively [43]. These results show that iron doped on the NC surface by the LPP method is in the form of iron oxide nanoparticles. C–O–C and C–O bonds were observed in the C 1s region. This may also be the carbon bond that the AC had from the beginning, or it could have been formed by the LPP reaction. In the N 1s region, peaks were observed at 399.3, 400.3, and 401.6 eV, which were assigned to *N*-pyridinic, *N*-pyrrolic/pyridonic, and graphitic nitrogen, respectively [44,45]. The LPP process shows that various nitrogen-containing functional groups are generated on the AC surface. Similar peaks in the N1s region were observed between NC and IONCC, which indicated the iron oxide nanoparticles generated from the LPP reaction did not affect nitrogen loaded on the AC surface. In the Fe 2p region, peaks were observed at 710.9 and 724.6 eV, which were assigned to Fe 2p_1/2_ and Fe 2p_3/2_, respectively. The spin orbital splitting (SOS) interval of Fe 2p_1/2_ and Fe 2p_3/2_ peaks was 13.6 eV. Therefore, the iron oxide nanoparticles synthesized by the LPP method are Fe_3_O_4_ [46]. The peaks at 710.9 and 712.8 eV in the Fe 2p_3/2_ region were attributed to Fe^2+^ and Fe^3+^, and are associated with the peaks at 724.3 and 726.7 eV in the Fe 2p_1/2_ region [47]. In addition, the peaks observed at 714.6 and 718.8 eV are the satellite peaks of Fe^2+^ and Fe^3+^, respectively, which are similar to previous reports [48,49]. When the chemical state of as-prepared composites as well as their chemical composition were reviewed by XPS, the elemental composition for IONCC-5, 10, and NC was in good agreement with that of EDS in FE-SEM (see Table 1).

**Figure 4:**
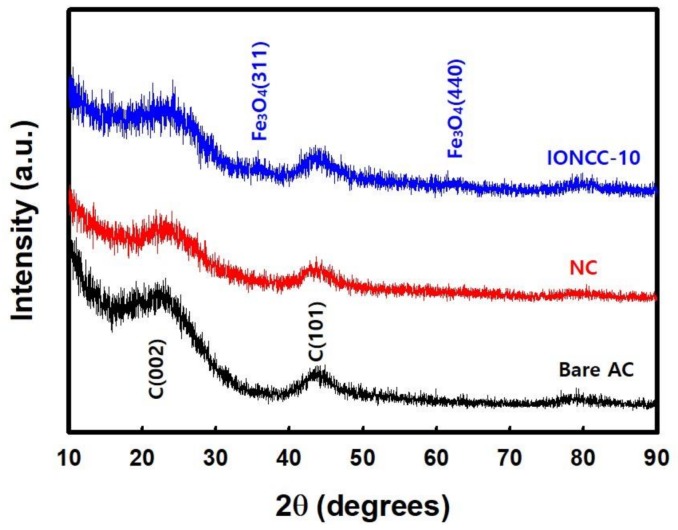
X-ray diffraction pattern of bare AC, NC, and IONCC-10.

Figure 4 presents XRD patterns of NC and IONCC-10 synthesized by the LPP process along with the pattern of AC. In the spectrum of bare AC, the 002 and 101 planes of carbon were observed at 24.5 and 43.9° 2θ. NC showed a similar pattern to bare AC but the peak of the 002 plane was broader, which was assigned to amorphous nitrogen doping of the AC surface by the LPP reaction [50,51]. As shown in Table 1, the content of iron oxide nanoparticles impregnated in IONCCs was less than 1 at. %, which was difficult to observe by XRD. On the other hand, in the IONCC-10 samples, the peaks for the 311 and 440 planes Fe_3_O_4_ were observed at 35.4 and 62.5° 2θ, respectively [52,53]. Therefore, the iron impregnated on the NC surface by the LPP reaction is iron oxide, which agrees with the EDS and XPS results.

**Figure 5:**
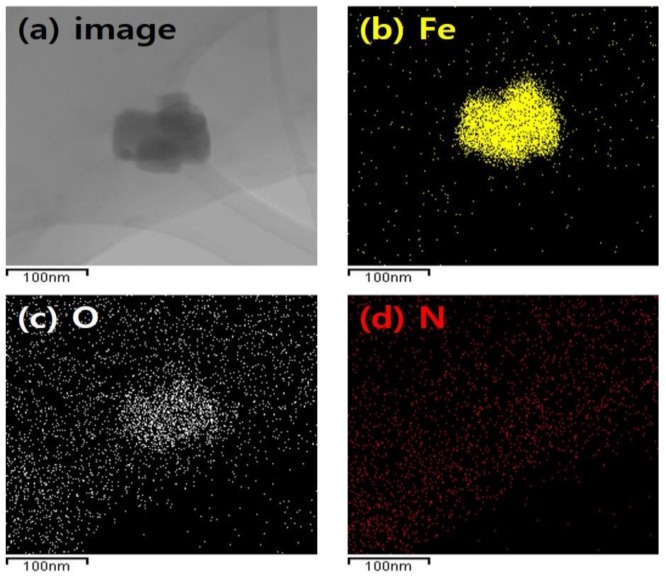
FE-TEM image and elemental mapped results of iron oxide nanoparticle on the IONCC-10; (**a**) TEM image; (**b**) iron; (**c**) oxygen; and (**d**) nitrogen element.

Iron oxide nanoparticles on the IONCC-10 synthesized by the LPP method was observed by FE-TEM, as shown in Figure 5, along with the elemental mapping image. The iron oxide nanoparticles produced by the LPP reaction had a size ranging from 40 to 100 nm. As shown in Figure 5a, approximately 40–100 nm of iron oxide nanoparticles were clustered on the IONCC surface. In the mapping images, iron, oxygen, and nitrogen were marked with yellow, white, and red dots, respectively. The particles on the NC surface were composed of iron and oxygen components. Oxygen was also found on the IONCC surface, which is consistent with the EDS and XPS results. On the other hand, nitrogen was distributed uniformly over the IONCC surface. The LPP reaction using the ammonium chloride reactant solution resulted in nitrogen being doped uniformly over the AC surface.

**Figure 6:**
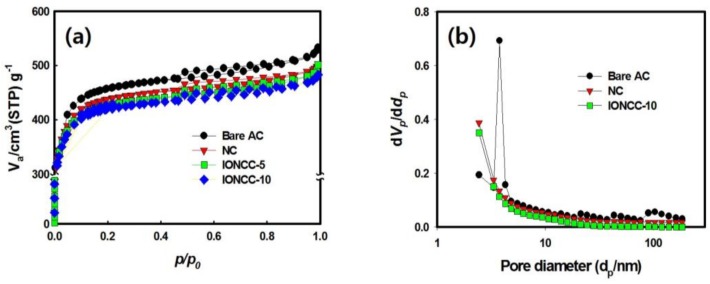
N_2_ adsorption-desorption isotherm curve (**a**) and pore size distribution (PSD) (**b**) of bare AC and as-prepared composites.

The effects of nitrogen doping and the impregnation of iron oxide nanoparticles on the surface area and pore diameter of AC were evaluated. Figure 6a shows the adsorption-desorption isotherm curves of N_2_ gas at 77 K for each sample. The hysteresis zone of the mesopore was observed in all samples and the hysteresis area of NC and IONCC decreased compared to bare AC. In the case of NC, the pores were blocked by the nitrogen generated on the AC surface in the LPP reaction, and the hysteresis area of the mesopore decreased [54,55]. In the case of the IONCCs, the N_2_ isotherm curves were affected by the iron oxide nanoparticles produced on the NC surface. Figure 6b presents the pore size distribution (PSD) measured by the Barrett, Joyner, and Halenda (BJH) method. Bare AC had a structure with developed mesopores, 2–5 nm in size. On the other hand, the pore size distribution of the mesopore tended to decrease in NC and IONACCs, as shown in Figure 6a, which is similar to the N_2_ isotherm curve.

Table 2 lists the surface area, total pore, and mean pore diameter measured using the Brunauer–Emmett–Teller (BET) method. The surface area and total pore volume of NC were smaller than those of bare AC and the mean pore size was also decreased. The micro- and mesopores are affected by the nitrogen generated on the AC surface by the LPP process and these values were reduced [42]. The surface area and total pore volume of IONCC-10 were smaller than IONCC-5 due to an increase in the amount of iron oxide nanoparticles added. This is because the proportion of iron oxide in the composite increases. In the case of the mean pore size, IONCCs were larger than NC. In addition, the mean pore size of IONCC-10 was larger than that of IONCC-5. The micro- and mesopore sizes of NC might have been blocked by the iron oxide particles produced by the LPP reaction.

### 3.2. Electrochemical Measurement

**Figure 7:**
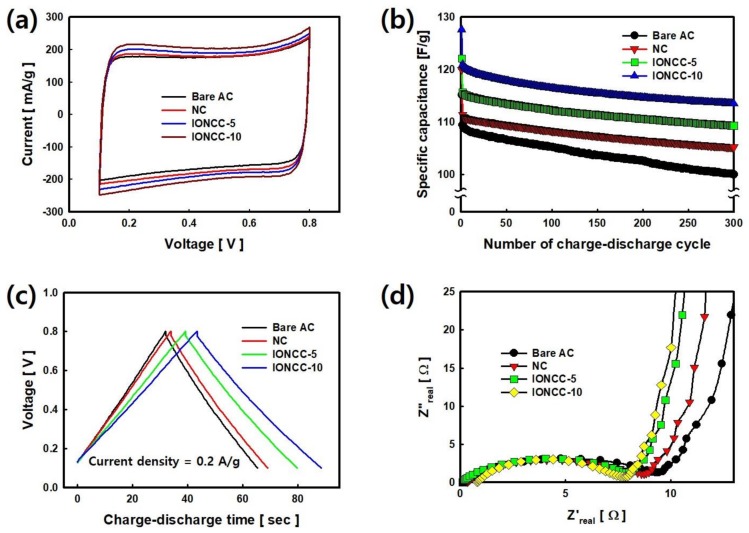
C–V curve (**a**); Cycling performance (**b**); V–t curve (**c**); and Nyquist plot (**d**) of bare AC and as-prepared composites using LPP method.

The electrochemical properties of NC and IONCCs synthesized by the LPP process were measured and compared with those of bare AC. The results are shown in Figure 7. Figure 7a presents the C–V curve results measured by CV over the range of 0.1 to 0.8 V, at a rate of 10 mV/s. Bare AC showed a typical rectangular shape and the characteristics of the EDLC. On the other hand, NC and IONCCs exhibited pseudo-capacitive behavior, and the area of the C–V curve was increased compared to that of bare AC. Nitrogen bonded to the surface of the NC reduced the surface area (see Table 2), but improved the faradic interactions with 6M KOH due to the improved wettability [56]. In addition, the C–V curve area tended to increase with increasing amount of iron oxide impregnated in IONCC because of the redox reaction by iron oxide [57]. Figure 7b shows the change in capacitance measured by repeating the charge–discharge process for 300 cycles. The initial specific capacitance of bare AC was 115.12 F/g, which decreased to 99.96 F/g after 300 cycles, showing 13.16% capacitance loss. The initial specific capacitance of NC was 119.98 F/g, which decreased to 105.18 F/g after 300 cycles, showing 12.33% capacitance loss. This has higher specific capacitance and stable cycling stability than that of bare AC because the hydrophilicity and reversibility of the redox reaction by nitrogen are increased [58]. The initial specific capacitance of IONCC-5 and IONCC-10 were 122.64 and 127.11, respectively. The specific capacitance increased with increasing amount of iron oxide impregnated by the LPP reaction, which can be observed as an increase in specific capacitance due to the redox reaction. The specific capacitance after the 300th cycle was 108.50 and 114.25 F/g, respectively, which showed a corresponding 11.52% and 10.11% loss ratio, indicating stable cycling stability compared to bare AC. Figure 7c presents a voltage-time (V–t) curve measured at a charge-discharge rate of 2 mA; the bare AC showed a typical symmetrical shape. Composites (NC, IONCCs) prepared by the LPP process exhibited pseudocapacitance behavior with a slight increase in the discharging process time. Figure 7d shows the results for a composite resistor measured over the range of 0.01 to 300 kHz. The semicircle in the high frequency region indicates the charge transfer resistance between the electrolyte and electrode, which is 9.18 Ω in the bare AC, and 8.41, 7.90, and 7.22 Ω in the NC, IONCC-5, and IONCC-10, respectively. The nitrogen content on the NC surface increased the compatibility and charge transfer rate. In the case of IONCCs, the conductivity of the electrode was improved by the iron oxide in the composites [59,60]. The slope in the low frequency region represents the capacitive behavior. Compared to the bare AC, the composites containing nitrogen and iron oxide exhibited pure capacitive behavior.

## 4. Conclusions

An electrochemical capacitor electrode was fabricated by doping with nitrogen and forming iron oxide nanoparticles on the AC surface using a LPP process. The following conclusions were obtained:

1. Nitrogen-doped carbon (NC) was prepared by a primary LPP reaction using an ammonium chloride reactant solution and nitrogen at a concentration of 3.77 at. % was formed uniformly over the AC surface.
2. Iron oxide/NC composite (IONCC) was prepared by a secondary LPP reaction using an iron chloride reactant solution. Iron oxide nanoparticles, 40–100 nm in size, were impregnated homogeneously over the NC surface, which were identified as Fe_3_O_4_ by XPS and XRD.
3. The amount of iron nanoparticles impregnated in the AC surface increased with increasing initial iron chloride concentration. The oxidation of AC and NC by the LPP reaction increased the oxygen content in the composites.
4. Bare AC exhibited a typical rectangular shape and the characteristic of EDLC. On the other hand, pseudo-capacitive behavior was observed in the NC and IONCCs, and the area of the C–V curve was greater than that of bare AC.
5. The nitrogen content on the NC surface increased the compatibility and charge transfer rate.
6. The impregnation of iron oxide nanoparticles on the NC by the LPP process improved the cycling stability of the EDLC and reduced the equivalent series resistance.

## Figures and Tables

**Table 1:** Chemical composition of bare AC and as-prepared composites using the LPP reaction with different initial iron precursor concentrations

Samples	Carbon		Oxygen		Nitrogen		Iron	
	wt. %	at. %	wt. %	at. %	wt. %	at. %	wt. %	at. %
Bare AC	96.13	97.06	3.87	2.94	0.00	0.00	0.00	0.00
NC	91.40	92.96	4.28	3.27	4.32	3.77	0.00	0.00
IONCC-5	88.94	92.21	4.83	3.76	3.96	3.52	2.27	0.51
IONCC-10	87.55	92.02	5.24	4.14	3.27	2.95	3.94	0.89

**Table 2:** Textural properties of bare AC and as-prepared composite obtained through the LPP process with different iron precursor concentrations

Sample	BET Surface Area (m^2^·g^−1^)	Total Pore Volume (cm^3^·g^−1^)	Average Pore Size (nm)
Bare AC	1700.7	0.8189	1.9261
NC	1592.6	0.7756	1.8328
IONCC-5	1547.7	0.7691	1.8534
IONCC-10	1504.5	0.7435	1.8670
